# The Most Prevalent Freeman-Sheldon Syndrome Mutations in the Embryonic Myosin Motor Share Functional Defects*

**DOI:** 10.1074/jbc.M115.707489

**Published:** 2016-03-04

**Authors:** Jonathan Walklate, Carlos Vera, Marieke J. Bloemink, Michael A. Geeves, Leslie Leinwand

**Affiliations:** From the ‡ School of Biosciences, University of Kent, Canterbury CT2 7NJ, United Kingdom and; the § Department of Molecular and Developmental Biology, University of Colorado, Boulder, Colorado 80309

**Keywords:** ATPase, enzyme kinetics, molecular motor, recombinant protein expression, skeletal muscle, human myosin, muscle disease, myosin subfragment 1, transient kinetics

## Abstract

The embryonic myosin isoform is expressed during fetal development and rapidly down-regulated after birth. Freeman-Sheldon syndrome (FSS) is a disease associated with missense mutations in the motor domain of this myosin. It is the most severe form of distal arthrogryposis, leading to overcontraction of the hands, feet, and orofacial muscles and other joints of the body. Availability of human embryonic muscle tissue has been a limiting factor in investigating the properties of this isoform and its mutations. Using a recombinant expression system, we have studied homogeneous samples of human motors for the WT and three of the most common FSS mutants: R672H, R672C, and T178I. Our data suggest that the WT embryonic myosin motor is similar in contractile speed to the slow type I/β cardiac based on the rate constant for ADP release and ADP affinity for actin-myosin. All three FSS mutations show dramatic changes in kinetic properties, most notably the slowing of the apparent ATP hydrolysis step (reduced 5–9-fold), leading to a longer lived detached state and a slowed *V*_max_ of the ATPase (2–35-fold), indicating a slower cycling time. These mutations therefore seriously disrupt myosin function.

## Introduction

Autosomal dominant mutations in 5 of the 10 human sarcomeric myosin heavy chain (MyHC)^4^ genes cause a wide variety of cardiac and skeletal myopathies (1), and new mutations continue to be discovered (2–5). There are two developmental myosin isoforms: MyHC-perinatal (*MYH8*) and MyHC-embryonic (*MYH3*), both of which are expressed primarily during fetal development and during muscle regeneration (6).

There have been few studies of developing muscle in humans; however, the order in which myosin isoforms are expressed has been described. Primary skeletal muscle fibers appear after 8 weeks of gestation, followed at 10 weeks by the appearance of secondary fibers, which become the predominant form by week 21 (7). Primary fibers express MyHC-emb and the cardiac or slow muscle isoform, MyHC-β (8, 9), with the MyHC-emb being detected before MyHC-β (10). Secondary fibers initially only express MyHC-emb, followed by MyHC-peri (10). At week 15, MyHC-emb accounts for about 81% of all human MyHC transcripts (11), whereas a population of tertiary fibers emerges at week 16 that expresses adult fast myosin (9, 12). Adult myosins and tertiary fibers gradually increase toward the end of gestation (9, 10) as both MyHC-emb and MyHC-peri are down-regulated, with MyHC-emb and MyHC-peri only being faintly detectable in a few fibers in 1-month-old infants. However, recently, MyHC-emb protein has been detected outside of this time window (13).

Mutations in MyHC-emb cause autosomal dominant distal arthrogryposis, the most severe form of which is Freeman-Sheldon syndrome (FSS) (11). Phenotypes include club foot, camptodactyly (contractures of the fingers or toes), contractures of oral facial muscles leading to a “whistling face” phenotype, and, in the most severe cases, scoliosis (14). There are currently eight identified MyHC mutations that cause FSS (14–16). 90% of FSS cases are linked to three mutations in the motor domain of MyHC-emb: R672C, R672H, and T178I (14). Patients who have the T178I mutation have the most severe phenotypes (facial contractures and congenital scoliosis), R672H results in intermediate severity, and R672C is the least severe (14). Adult muscle fibers from individuals with wild type MyHC-emb and those with the R672C mutation do not differ in expression levels of MyHC-emb protein (13). Tajsharghi *et al.* (15) presented data from an FSS patient with a T178M mutation in *MYH3*. At 15 months, the individual had a higher level of MyHC-peri expression (>20% of all fibers) compared with that of eight “healthy” control samples (0–2% of all fibers) taken at a similar time of development (10–15 months). Samples taken from the same patient at 5 years showed a predominance of type-1/MyHC-β expression with small, scattered type-1 fibers. This suggests that there could be a compensatory effect or a developmental defect that causes MyHC-peri to be expressed later or longer in postnatal development. Tajsharghi *et al.* (15) hypothesized that the myopathy occurs during embryonic development and that after birth, when MyHC-emb is down-regulated, there is a recovery of the muscle leaving some residual muscle defects. Functional studies on muscle fibers from healthy and FSS individuals with the R672C mutation showed that the mutant fibers had reduced force, prolonged relaxation time, and incomplete relaxation (13).

Embryonic myosin heavy chain is the most abundant during development, when muscles are being formed (6). Because distal muscles are more affected in FSS, it is interesting to note that distal muscles appear to be formed earlier during development than proximal muscles (17). They may therefore express embryonic myosin for a longer period of time than proximal muscles.

**FIGURE 1.**
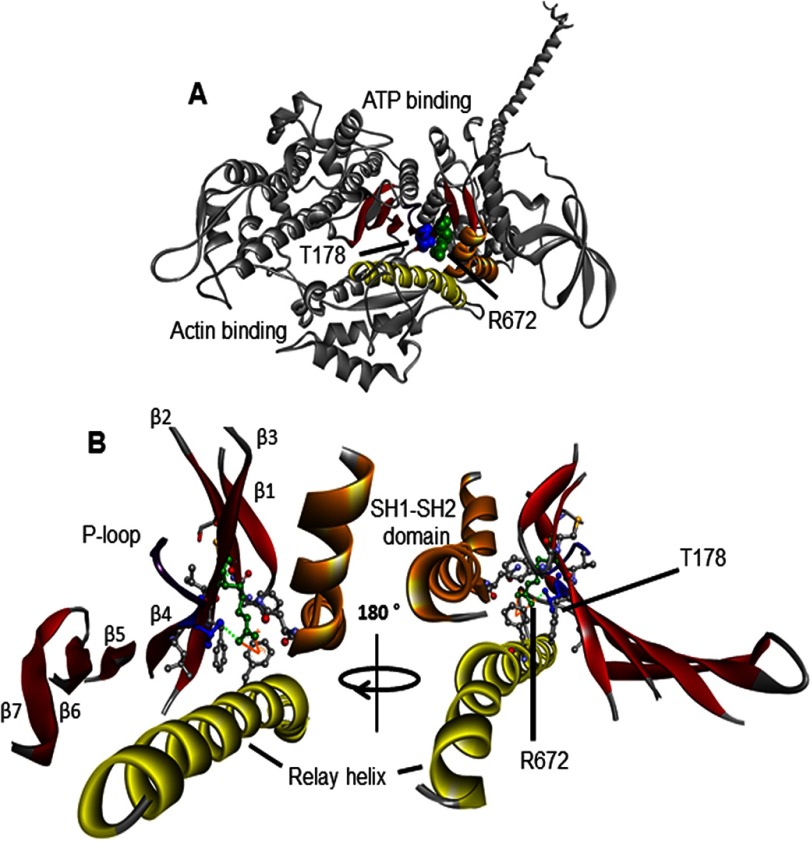
**Homology model of embryonic myosin S1.** This *ribbon structure* is based on the scallop structure in the pre-power stroke conformation (PDB code 1QVI). *A*, the myosin heavy chain is shown in *gray*, the SH1-SH2 helices in *orange*, the relay helix in *yellow*, the P-loop in *purple*, and the seven-stranded β-sheet in *red*. The arginine 672 (*R672*) and threonine 178 (*T178*) residues are shown in *green* and *blue space filling mode*, respectively. Both of these residues are highly conserved across species and myosin isoforms, including the scallop myosin and human MyHC-emb. Both residues are in the center of the S1 domain located in the upper 50-kDa domain near the ATP binding cleft. *B*, *enlargement* of the region around Arg^672^ (located on the third β-strand) and Thr^178^ on the fourth β-strand of the seven-stranded β-sheet that runs through the S1 domain. Thr^178^ is also at the base of the P-loop (GESGAG, residues 179–184), which is involved in ATP binding. The *green dotted line* represents hydrogen bonds that can be seen between Thr^178^ and Arg^672^, whereas the *solid orange line* indicates a π-cation bond that can be seen between Arg^672^ and Phe^490^. The interactions of Arg^672^ and Thr^178^ are summarized in Tables 2 and 3. Residues not labeled are those found to interact with either Arg^672^, Thr^178^, or both and are summarized in Tables 2 and 3.

To understand the pathogenesis of FSS, it is necessary to understand the nature of the defect in the mutant myosin motor proteins and how it might be corrected. Both residues involved, Thr^178^ and Arg^672^, are highly conserved in all myosin II molecules and located near the ATP binding site close to the P-loop, where the γ-phosphate of ATP binds (Fig. 1). Examination of homologous myosin motor domain structures (subfragment 1 or S1; Fig. 1*A*) shows that Thr^178^ is at the end of the fourth strand of the central seven-stranded β-sheet and close to the ATP binding site at the base of the P-loop (Fig. 1*B*) in a location that can influence ATP binding and hydrolysis. Arg^672^ is located on the third β-strand with the side chain buried, which interacts with the relay helix, SH1-SH2 domain, and the β-strands on either side, regions that go through major conformational changes during the myosin cross-bridge cycle (18, 19). Notably, the crystal structures show an interaction between Arg^672^ and Thr^178^ that may account for the similar phenotypes in the mutants.

Due to the difficulty of isolating homogeneous myosin samples from human muscle and the limited ability to obtain samples from FSS patients, we utilized an established recombinant expression approach (20). This approach was first used by Wang *et al.* (21), and we have used it to study the kinetics of all human adult skeletal and cardiac isoforms and two mutations in the human cardiac MyHC-β associated with hypertrophic cardiomyopathy (22–24). These studies have demonstrated that this is a powerful technique for studying homogeneous, human, wild type, and mutant myosin S1 samples using both biochemical kinetics and *in vitro* motility approaches. We used this recombinant approach to study pure human WT MyHC-emb and the three FSS-causing mutants. Although some results have been reported on embryonic and R672C mutant muscle fibers (11, 13), to our knowledge, this is the first detailed functional study of purified WT MyHC-emb and any of the mutations that cause FSS.

## Materials and Methods

### 

#### 

##### Proteins

Human embryonic MyHC-S1 proteins were expressed and purified as described previously (20). We constructed shuttle plasmids containing the S1 coding region for *MYH3* (Met^1^–Ser^843^) upstream of a His_6_ tag. Using the pAdEasy kit, these plasmids were used to construct recombinant replication-deficient adenovirus that express *MYH3*. We first cloned the WT *MYH3* gene and then performed site-directed mutagenesis to produce the R672C, R672H, and T178I mutations. The viral particles were amplified using HEK293 cells, the cell lysates were clarified using cesium chloride gradients, and the concentrated virus was stored in a glycerol buffer at −20 °C.

Some alterations were made to the published expression system. For cell culture, we used the four-layer Nunc^TM^ Cell Factory system to increase culturing capacity. As described previously (20) for expressing S1 in this system, C_2_C_12_ cells need to be differentiated from myoblasts to myotubes. The definition of multiplicity of infection does not apply with a syncytial multinuclear cellular structure combined with a larger culturing surface area, which results in an indeterminate cell count. To overcome this and standardize our infection, we used an *A*_260_ measurement at a 1:100 dilution and a conversion factor of 1 *A*_260_ = 1.1 × 10^12^ viral particles/ml (25, 26). For example, for an *A*_260_ of 0.1, we would infect a 100-mm dish with 10 μl of concentrated virus. When extrapolating to a four-layer cell factory, we took into account the surface areas of a 100-mm dish and a four-layer cell factory.

**FIGURE 2.**
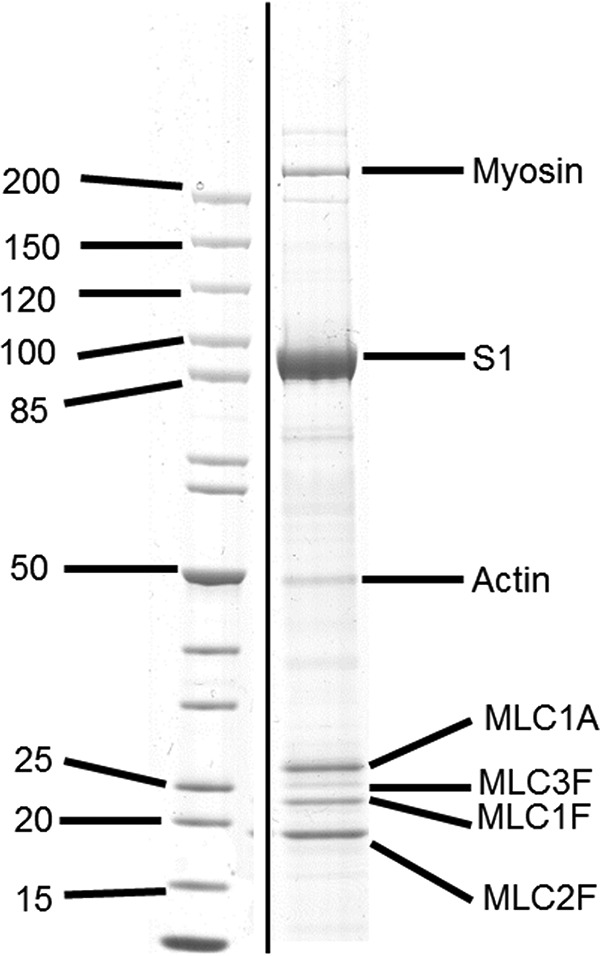
**SDS-PAGE of purified wild type embryonic S1.** The myosin S1 co-purifies with a small amount of endogenous mouse full-length myosin (<5% by weight) and the four mouse light chains: three major bands (MLC2F (regulatory), MLC1F, and MLC1A (both essential)) and a minor band (MLC3F). The three major bands have been identified in embryonic S1 (25), suggesting that the fourth light chain (MLC3F) is binding to the endogenous myosin.

Infected C_2_C_12_ cells were incubated for 5 days and frozen into a cell pellet. This was then homogenized in a low salt buffer and centrifuged, and the supernatants were purified by two procedures. For stopped flow, we used nickel affinity chromatography (HisTrap HP 1-ml column). Afterward, the S1 was dialyzed into the low salt stopped-flow buffer (25 mm KCl, 20 mm MOPS, 5 mm MgCl_2_, 1 mm DTT, pH 7.0, Fig. 2). From between 1500 and 3000 cm^3^ of cultured C_2_C_12_ cells, we isolated 1–2 mg of purified MyHC with a concentration between 10 and 20 μm. A small amount of endogenous myosin persisted in the S1 sample purified for stopped flow. Using densitometry software (Scion Image) to analyze SDS-polyacrylamide gels, we calculated the contaminate myosin as <5% of the S1 by mass, or a 1:40 molar ratio of myosin/S1. It is possible that the myosin and S1 were bound to actin and so were co-purified.

For ATPase measurements, the purification scheme was modified and extended. Nickel affinity chromatography was performed by gravity in a Bio-Rad Econo-Pac column packed with HisPur nickel-nitrilotriacetic acid resin. Eluates were dialyzed in a low salt/low imidazole buffer. The eluted material was subjected to anionic exchange chromatography (HiTrap HP 1-ml column) and desalted by another buffer exchange into the ATPase assay buffer (see the ATPase method). This gave a higher purity of S1 necessary for ATPase assays.

As reported previously (20), the recombinant S1 co-purifies with the endogenous mouse light chains (Fig. 2). Essential light chains MLC1A and MLC1F and regulatory light chain MLC2F appear to purify with the recombinant S1 because their bands are much denser than the essential light chain MLC3F and in the correct ratio to the heavy chain. They are the same light chains that have been found to associate with embryonic myosin in tissue (25). The percentage identity between the human and mouse light chains is 90–95%. Rabbit skeletal actin was prepared as described previously (27) and labeled with pyrene at Cys^374^ (28).

##### ATPase

For steady-state ATPase measurements, we modified the NADH-coupled assay as described (29). This assay system couples the ADP production to NADH oxidation to NAD^+^; therefore, absorbance was read at 340 nm. We used a 96-well format, and the assay buffer used in all experiments was 12 mm PIPES, 2 mm MgCl_2_, and 1 mm DTT at pH 6.8 and 25 °C. Bovine cardiac actin was purified as described previously (27) and used for ATPase measurements. Once thoroughly and rapidly mixed, the assays were read for 30 min in a Molecular Devices SpectraMax reader to monitor absorbance over time. The Michaelis-Menten equation was fit to the data to determine the maximal activity (*V*_max_) and the associated actin constant for myosin (*K_m_*) using GraphPad Prism.

##### Transient Kinetics

**SCHEME 1.**
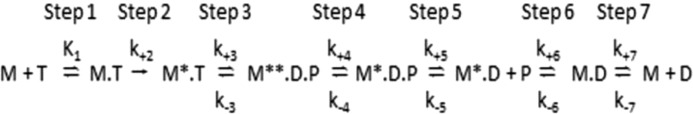
**Seven-step scheme of ATP binding to myosin.**

All kinetic experiments were conducted in 20 mm MOPS buffer with 25 mm KCl, 5 mm MgCl_2_, and 1 mm DTT, pH 7, at 20 °C, unless otherwise indicated. Measurements were performed with a High-Tech Scientific SF-61 DX2 stopped-flow system. The concentrations stated are those after mixing in the stopped-flow observation cell unless otherwise stated. All stopped-flow traces were analyzed in either software provided by TgK Scientific (Kinetic Studios) or Origin (Microcal). Intrinsic tryptophan fluorescence was measured by excitation at 295 nm and observed through a WG320 filter. In the absence of actin, the kinetics of S1 and ATP or ADP were interpreted using the seven-step model described by Bagshaw *et al.* (30) (Scheme 1), where the forward rate constants are *k*_+_*_i_* and the reverse rate constants are denoted k_−_*_i_. K_i_* = *k*_+_*_i_*/*k*_−_*_i_* representing the equilibrium constant at the *i*th step in the reaction.

In the ADP displacement from S1 assays, the two amplitudes are proportional to the concentration of S1 present as either free S1 or S1-ADP. ADP concentrations determine the amplitudes as follows.

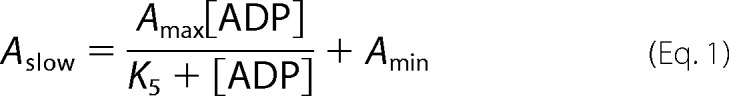


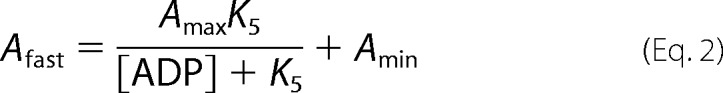


**SCHEME. 2.**
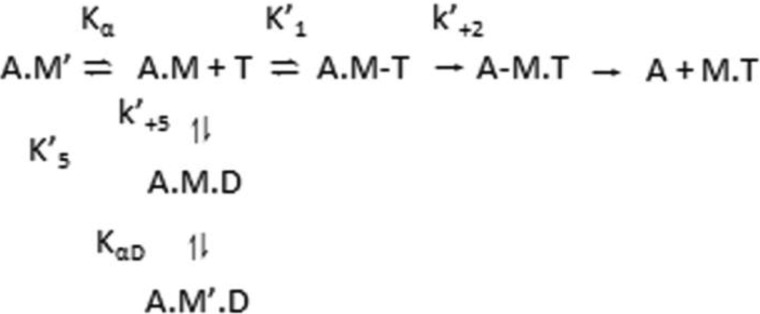
**ATP or ADP binding to actomyosin leading to the dissociation of myosin from actin.**

When actin was present, the sample was excited at 365 nm, and emission of pyrene-labeled actin was detected after being passed through a KV399 cut-off filter. The binding of S1 to pyrene-labeled actin quenches the fluorescence; therefore, dissociation could be measured by an increase in fluorescence. The interactions between actin-S1 and ATP or ADP were analyzed based on the model in Scheme 2 (22).

Following a reversible binding of ATP to actomyosin, there is a rate-limiting isomerization (*k*′_+2_) of the complex, leading to rapid actomyosin dissociation. ADP and ATP compete for the nucleotide binding site on the S1, and ADP binding is governed by the dissociation constant *K*′_5_ (= *k*′_+5_/*k*′_−5_).

A dissociation reaction can be measured when the pyrene-labeled actin is in a complex with S1 because the complex quenches the pyrene signal. ATP induces the dissociation of S1 from actin, resulting in an increase in fluorescence, allowing the reaction kinetics to be determined. Using Scheme 2, Equation 3 can be used to determine the constants *K*′_1_*k*′_+2_, *k*′_+2_, and 1/*K*′_1_.

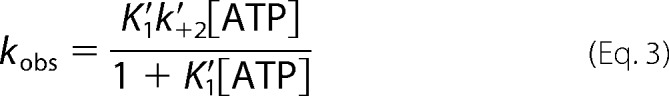


Equation 4 defines the kinetics when ATP and ADP are in rapid competition for binding to the actomyosin complex, and *K*′_1_ [ATP] < 1, such that the equation is linear with respect to [ATP].

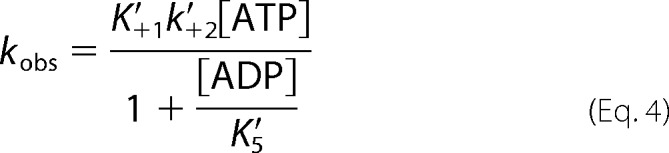


If, in the absence of ADP, *k*_obs_ = *k*_0_, then normalizing Equation 4 can make different myosin isoform comparisons easier.

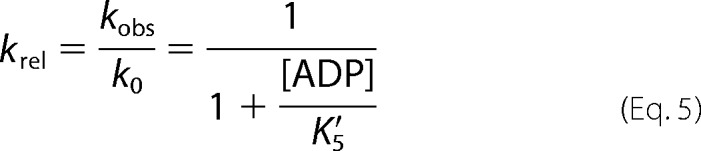


ADP release (*k*′_+5_) was determined by the *k*_obs_ when the saturating ADP was incubated with actin-S1 and rapidly mixed with high concentrations of ATP.

To determine the actin affinity, an S1 titration assay was performed as described by Kurzawa and Geeves (31). Fixing the concentration of pyrene-labeled actin, the amplitude of the ATP-induced dissociating reaction provides an estimate of the fraction of actin bound to S1 at increasing S1 concentrations. The amplitude dependence of the S1 concentration was fitted to the physically significant root of the following quadratic equation.

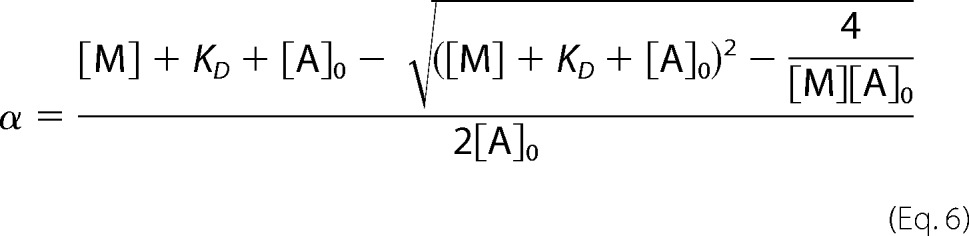


In all cases, the data in the figures refer to individual experimental measurements, whereas Table 1 gives mean values of the fitted constant for a minimum of three separate measurements using different protein preparations.

##### Sequence Alignments and Homology Modeling

Three-dimensional homology models were generated (Fig. 1) for the human embryonic isoform using the SWISS-MODEL automatic comparative protein modeling server (32–35). The primary sequence for the embryonic myosin was aligned pairwise with the sequence of the six scallop structures (PDB codes given below) using the Clustal Omega alignment protocol. These alignments were submitted to SWISS-MODEL, which generated the models. The sequence identity between the human embryonic S1 sequence and the scallop myosin was 60%, which allowed us to build well resolved structures. The scallop structures were used because they represent different conformational states of the cross-bridge cycle: post-rigor-like structure with ADP bound (PDB code 2OTG), a rigor-like structure without nucleotide bound (PDB code 2OS8), a post-rigor structure without a nucleotide bound (PDB code 1SR6), a pre-power stroke state containing ADP-VO_4_ (PDB code 1QVI), the actin-detached state containing ADP-BeF_x_ (PDB code 1KK8), and a near rigor state with ADP bound (PDB code 1S5G). We compared the interactions we found in the homology to the two MyHC-β structures (4P7H and 4DB1, sequence identity 79%) to validate their existence in human myosin structures as well as the original scallop structures.

## Results

### 

#### 

##### Nucleotide Binding to Actin-S1 and Actin Binding to S1

The detachment of the actin-myosin cross-bridge is the step that is thought to limit shortening velocity in contracting muscle fibers. This can be limited by either the rate of ATP binding to the cross-bridge or the preceding ADP release step (Scheme 2). Because these are the first reports of detailed kinetics of wild type (WT) MyHC-emb, we will compare its properties to another slow myosin, the well characterized MyHC-β. For slow type myosins, the affinity of ADP for actin-S1 is normally tighter than the fast type myosins (IIa, IIb, IIx, α), and ADP release limits the detachment and the overall muscle shortening velocity (36–38). Therefore, we examined these steps first.

##### ATP-induced Detachment from Actin Is Slower for Myosin S1 Carrying FSS Mutations

**FIGURE 3.**
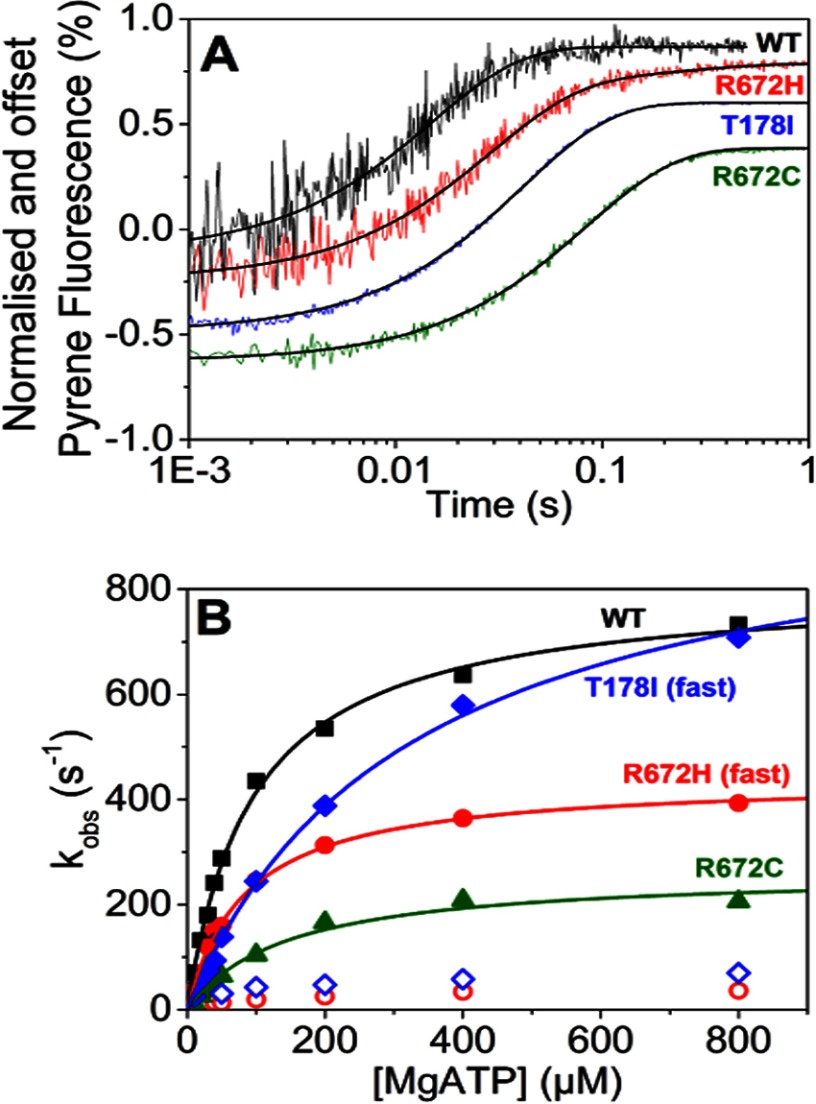
**ATP-induced dissociation of embryonic S1 from pyrene-labeled actin.** *A*, traces of 50 nm WT emb-S1 and the three mutants preincubated with equimolar pyrene-labeled actin and then rapidly mixed with 10 μm ATP. Each trace has been normalized and offset by the previous one by 0.2% fluorescence. At 10 μm ATP, WT MyHC-emb, R672C, and T178I were best described by a single exponential fit resulting in a *k*_obs_ = 70 s^−1^ (amplitude = 27%), 12 s^−1^ (amplitude = 46%), and 24 s^−1^ (amplitude = 45%) for WT MyHC-emb, R672C, and T178I, respectively. R672H was best described by a double exponential resulting in a fast and slow phase, giving *k*_obs_ = 36 s^−1^ (amplitude = 32%) and *k*_obs_ = 4.4 s^−1^ (amplitude = 3.8%), respectively. At all [ATP], WT and R672C were best described by a single exponential, as was T178I at [ATP] <50 μm. At ≥50 μm ATP, the T178I mutation was best described by a double exponential, whereas the R672H had a double exponential transient at all [ATP]. *B*, the dependence of *k*_obs_ on [ATP] yielded *K*′_1_*k*′_+2_ = 8.5, 5.2, 1.8, and 3.1 μm^−1^ s^−1^ for WT MyHC-emb (*black filled squares*), R672H (*red filled circles*), R672C (*green filled triangles*), and T178I (*blue filled diamonds*). The maximum rate yielded a *k*′_+2_ = 806 s^−1^ for WT MyHC-emb; 439 and 36 s^−1^ for the R672H fast and slow (*red open circles*) phases, respectively; 262 s^−1^ for R672C; and 1007 and 49 s^−1^ for the T178I fast and slow (*blue open diamonds*) phases, respectively. Values given here are for a single assay.

The ATP-induced dissociation of S1 from actin was monitored by the fluorescence of a pyrene label covalently attached to Cys^374^ of actin (Fig. 3*A*). At low concentrations of ATP, WT MyHC-emb, R672C, and T178I transients are best described by a single exponential fit. At all ATP concentrations, there is a second slower phase for R672H, which is best described by a double exponential fit. At high ATP concentrations ([ATP] > 100 μm), T178I develops a slow second phase, similar to the slow component in R672H. A slow component was not seen for the WT MyHC-emb or the R672C at any concentration of ATP used. At low ATP concentrations, there is a linear relationship between *k*_obs_ and ATP concentration for all constructs (Fig. 3*B*), which gives a second order rate constant of actin dissociation (*K*′_1_*k*′_+2_; Table 1). Compared with β-cardiac myosin S1, the WT MyHC-emb shows faster ATP-induced dissociation kinetics, whereas for the FSS mutants, this process is slower (Table 1 and see Fig. 9).

**TABLE 1:**
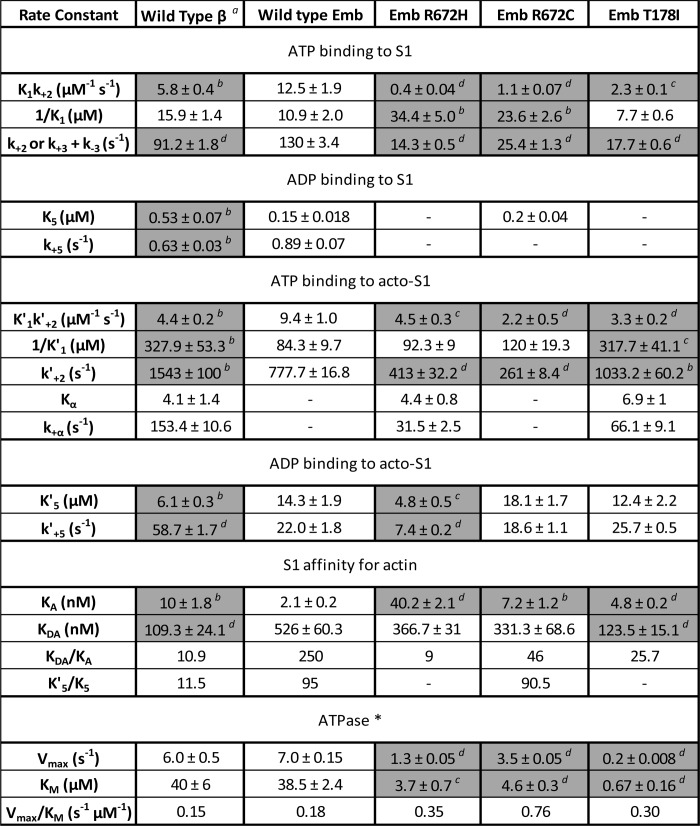
**Transient kinetic parameters measured for wild type β and embryonic, R672H, R672C, and T178I myosin S1 mutations** The values represent the mean ± S.E. based on a minimum of three different protein preparations. All measurements were performed at 20 °C in 25 mm KCl, 20 mm MOPS, 5 mm MgCl_2_, 1 mm DTT (pH 7) unless stated otherwise. Shaded boxes show the values that have changed significantly.

* KCl concentration 0 mm.

*^a^* Nag *et al.* (24) in same ionic strength as this experiment obtained from C2C12 cells.

*^b^ p* < 0.05 determined by Student's *t* test as compared with wild type embryonic S1.

*^c^ p* < 0.01 determined by Student's *t* test as compared with wild type embryonic S1.

*^d^ p* < 0.001 determined by Student's *t* test as compared with wild type embryonic S1.

##### The Maximum Rate of ATP-induced Dissociation Is Significantly Altered by the FSS Mutations

Over a wide range of ATP concentrations, the relationship between *k*_obs_ and ATP concentration is best described by a hyperbola (Fig. 3*B*), which gives a maximum rate constant of dissociation (*k*′_+2_) and the affinity of ATP binding in the initial step, 1/*K*_1_. There is a second slow component found at all ATP concentrations for R672H and at the higher ATP concentration (>100 μm) for T178I. One cause of a slow component could be ADP contamination; therefore, S1 was incubated with apyrase to hydrolyze any ADP. However, this has no effect on the slow component for either the R672H or T178I. This led us to the hypothesis that the slow component is caused by a conformational change in the ATP binding pocket from a closed to open state (the equilibrium constant for this conformational change, *K*_α_; see Scheme 2) previously seen in MyHC-β (39). Values for *K*_α_, calculated from the ratios of the fluorescent amplitudes for both components, and the maximum rate constant (*k*_+α_) are summarized (Table 1).

##### ADP Affinity (K′_5_) for WT MyHC-emb Motor Domain and the Three Mutations Is Typical of a Slow Myosin

**FIGURE 4.**
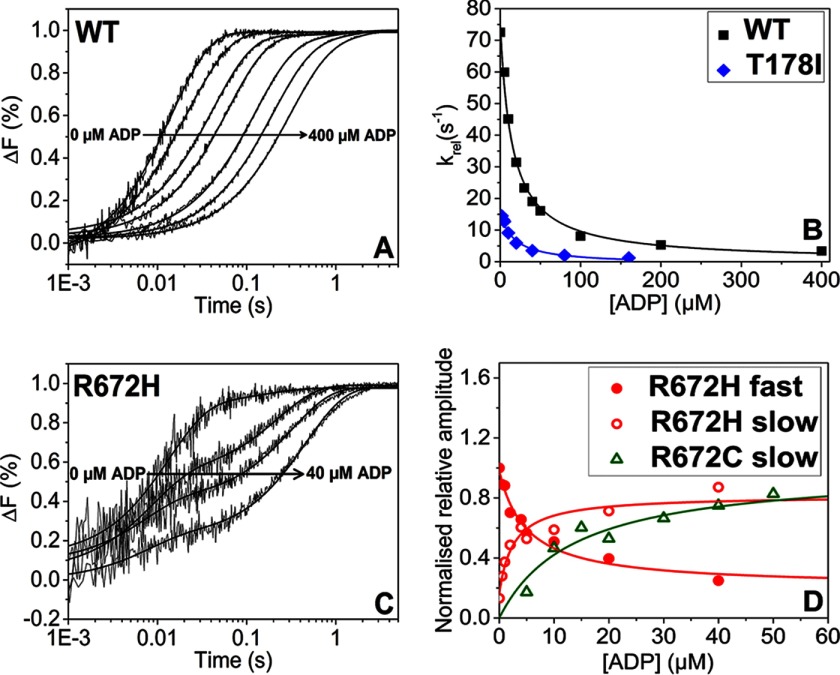
**ADP affinity for pyrene-labeled actin-S1.** *A*, fluorescent traces of 50 nm pyrene-labeled actin preincubated with 50 nm WT MyHC-emb and increasing [ADP] (0–400 μm) rapidly mixed with 10 μm ATP. As the [ADP] increased, the *k*_obs_ decreased from 72 to 3 s^−1^. *B*, the dependence of *k*_obs_ on [ADP] for WT MyHC-emb (*squares*) and T178I (*circles*), resulting in a *K*′_5_ = 16 ± 1.5 and 17 ± 4 μm, respectively. *C*, traces of 50 nm pyrene-actin preincubated with 50 nm R672H S1 and increasing [ADP] (0–40 μm) rapidly mixed with 30 μm ATP. Because there was a consistent slow phase even at low [ATP], the rates stayed constant while the amplitude of the fast phase decreased from 26 to 6% and the slow phase amplitude increased from 3.5 to 21%. This behavior was also seen in the R672C mutation. *D*, dependence of fluorescence amplitude of [ADP] for R672H and R672C. *K*′_5_ = 2.6 ± 1.1 μm for the fast phase and *K*′_5_ = 5 ± 1.3 μm for the slow phase for R672H. *K*′_5_ = 16.3 ± 3.6 μm for the fast phase (not shown) and 13.6 ± 2.6 μm for the slow phase.

To determine ADP affinity in the presence of actin, a competition assay was used (Fig. 4*A*). The observed rate constant (*k*_obs_) reduces as ADP increases and exhibits a hyperbolic dependence on ADP concentration defining the ADP affinity, *K*′_5_ (Fig. 4*B*). A similar measurement for T178I results in a value of *K*′_5_ that is not significantly different from that for WT MyHC-emb. The ADP affinity for WT MyHC-emb is about 2-fold weaker than the WT β-myosin S1. The FSS mutation T178I does not alter ADP affinity compared with WT MyHC-emb.

The hyperbolic dependence of *k*_obs_ on ADP concentration, found for WT MyHC-emb and T178I, is consistent with the rate constant of ADP release being of the same order or faster than the rate constant for ATP binding (*K*_1_*k*_+2_[ATP]) under these conditions. This was confirmed by measuring the rate of ADP displacement directly by incubating the S1 with a much higher ADP concentration and displacing with several high ATP concentrations (millimolar) (39). The trace can be best described by a single exponential signifying ADP release from S1 and ATP binding. The *k*_obs_ is unaffected by [ATP] and therefore remains constant. The ADP release rate constant is not significantly different from WT MyHC-emb. The ADP release rates for WT MyHC-emb are significantly slower compared with cardiac β-myosin (Table 1). The ADP release rate constant for WT MyHC-emb was significantly slower than cardiac β-myosin.

This type of hyperbolic dependence on ADP concentration is not found for the other two mutations (R672H and R672C), which shows a biphasic transient (Fig. 4*C* shows R672H as an example). This is consistent with the rate constant for ADP release (*k*′_+5_) being slower than the rate constant for ATP binding (R672H). Whereas the *k*′_+5_ of R672C is similar to that of the WT, the *k*′_+2_ is much slower, leading to the biphasic nature. The fast phase results from ATP binding to the fraction of actin-S1, which is free of ADP, whereas the slow phase arises from the fraction of myosin as actin-S1-ADP, from which ADP must first dissociate before ATP can bind.

For R672H, the observed rate constant of the slower phase remains constant, whereas the amplitude increases at increasing ADP concentration. In contrast, the fast phase amplitude decreases, whereas the observed rate constant also remains constant (Fig. 4*C*). A similar behavior was also seen for the R672C mutation. When the fast and slow phase amplitudes are plotted against ADP concentration, both could be described by a hyperbolic function (Fig. 4*D* shows both phases for R672H and the slow R672C phase), giving an overall ADP affinity (*K*′_5_). Comparing these three FSS mutants with WT MyHC-emb, the ADP affinity of R672H is about 3-fold tighter, whereas for R672C and T178I, this parameter is not significantly affected (Table 1).

##### FSS Mutations Reduce the Affinity of Actin for Myosin S1 but Increase Actin Affinity in the Presence of ADP

**FIGURE 5.**
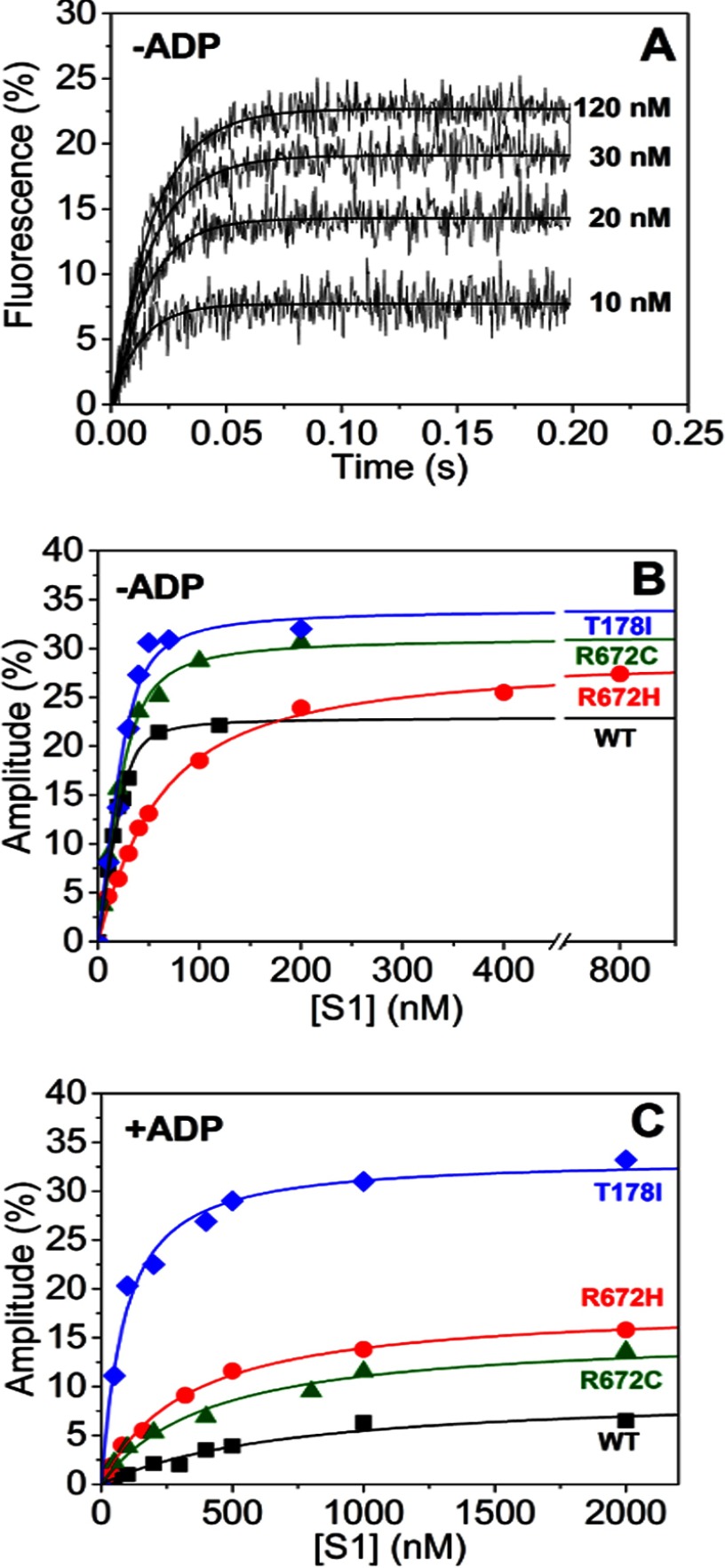
**Embryonic S1 affinity for actin in the absence and presence of ADP.** *A*, traces of increasing concentrations (0 μm to 120 nm) of WT MyHC-emb preincubated with 30 nm pyrene-labeled actin and then rapidly mixed with 10 μm ATP. Over a concentration series, the fluorescence amplitude increased with [S1]. *B*, the dependence of amplitude on [S1] can be described by a quadratic function (Equation 6), giving a *K*_A_ value of 2.5 nm for WT MyHC-emb (*filled squares*), 43 nm for R672H (*open squares*), 6.1 nm for R672C (*filled circles*), and 5.2 nm for T178I (*open circles*). *C*, repeating the same experiment but this time incubating the actin-S1 with saturating (20 × *K*′_5_) [ADP]. Plotting the amplitude against the [S1] again gives a quadratic dependence, which in turn gives a *K*_DA_ value of 706 nm for WT MyHC-emb (*filled squares*), 306 nm for R672H (*open squares*), 386 nm for R672C (*filled circles*), and 71 nm for T178I (*open circles*). Concentrations of S1 are before mixing.

By preincubating 30 nm pyrene-labeled actin (concentration before mixing) with varied S1 concentrations and then rapidly mixing with 20 μm ATP (concentration before mixing; Fig. 5*A*), it is possible to determine actin affinity (*K*_A_; Fig. 5*B*). This measurement was repeated for the three FSS mutations. R672C and T178I have a similar *K*_A_ value as WT MyHC-emb, whereas R672H has an affinity almost 20-fold weaker compared with WT MyHC-emb (Table 1).

This assay was repeated with a constant saturating ADP concentration (20 times the *K*′_5_ value) preincubated with the actin-S1 mixture to determine the actin affinity in the presence of ADP (*K*_DA_). The affinity of the WT MyHC-emb for actin weakens 250-fold (Fig. 5*C*). All of the mutants, however, have an affinity 2–4-fold tighter than the WT (Table 1).

##### Nucleotide Binding to Myosin S1 in the Absence of Actin

In the absence of actin, ATP binding to myosin can be monitored by a change in the intrinsic tryptophan fluorescence. The fluorescence change is caused by structural changes induced by both ATP binding and the ATP hydrolysis step (Scheme 1, *steps 1–3*). Although the binding of ATP to myosin is not part of the working cross-bridge cycle, the hydrolysis step controls how long myosin remains detached from actin.

##### The FSS Mutations Reduce the ATP Binding and Hydrolysis Rates for MyHC-emb S1

**FIGURE 6.**
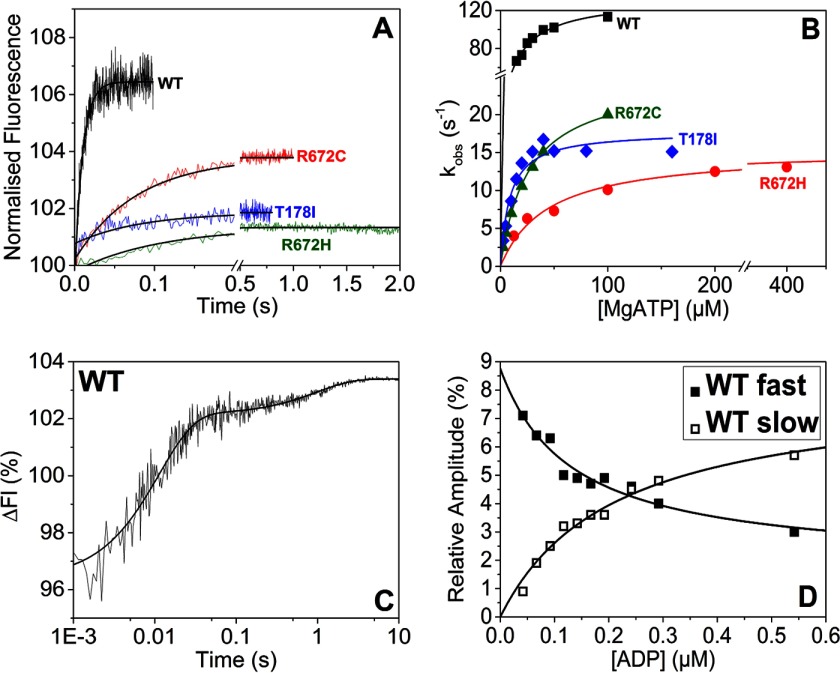
**Nucleotide binding to embryonic S1.** *A*, tryptophan fluorescence changes observed on rapidly mixing 50 μm ATP with 100 nm WT MyHC-emb and for the three mutants S1s. All four were best described with a single exponential fit yielding *k*_obs_ = 102 s^−1^ (amplitude = 6.6%), 7.3 s^−1^ (amplitude = 1.6%), 12 s^−1^ (amplitude = 3.5%), and 18 s^−1^ (amplitude = 1.4%) for WT MyHC-emb, R672H, R672C, and T178I, respectively. The single exponential fits are shown with *solid black lines. B*, the hyperbolic dependence of *k*_obs_ on [ATP] yielded *K*_1_*k*_+2_ = 8.8, 0.3, 0.95, and 2.1 μm^−1^ s^−1^ for WT MyHC-emb (*filled squares*), R672H (*open squares*), R672C (*filled circles*), and T178I (*open circles*), respectively. The maximum rate gives a value of *k*_max_ = 134 s^−1^ for WT MyHC-emb, 15.2 s^−1^ for R672H, 25 s^−1^ for R672C, and 17.8 s^−1^ for T178I. *C*, the protein fluorescence observed after mixing 0.1 μm WT MyHC-emb preincubated with 50 nm ADP with 50 μm ATP. The data could be best described by a double exponential function with a fast phase (*k*_obs_ = 92 s^−1^ and amplitude = 5.9%) and a slow phase (*k*_obs_ = 1 s^−1^ and amplitude = 1.3%). *D*, the dependence of the amplitudes of the fast and slow phases on [ADP] was hyperbolic, resulting in a *K_D_* = 0.13 ± 0.06 μm for the fast phase (*filled squares*) and *K_D_* = 0.12 ± 0.03 μm for the slow phase (*open squares*). This measurement was not possible with the R672H or T178I mutations, whereas there was only one phase for the R672C.

The binding and hydrolysis of ATP by myosin was observed via the intrinsic fluorescence of S1. S1 at 100 nm was mixed with varying concentrations of ATP (Fig. 6*A*).

All three FSS mutants have significantly slower *k*_obs_ values and smaller amplitudes than WT MyHC-emb. The amplitudes are greatly reduced in the R672H and T178I mutations, and increasing the S1 concentration does not increase the amplitude for either of these two mutants. The second order rate constant of ATP binding, *K*_1_*k*_+2_, was determined by varying the ATP concentration and plotting *k*_obs_ as a function of ATP (Fig. 6*B*). ATP binding for WT MyHC-emb is about 2-fold faster than for β-myosin S1, and all three mutants have significantly lower second order rate constants compared with WT MyHC-emb (Table 1). The three FSS mutations strongly reduce ATP binding to myosin S1 compared with WT MyHC-emb.

The maximum rate of ATP binding for WT MyHC-emb (*k*_max_) was also determined from the hyperbolic fits (Fig. 6*B* and Table 1). The value of *k*_max_ can either be attributed to the maximum rate of ATP binding (*k*_+2_) with switch 1 and switch 2 closing or the hydrolysis step (*k*_+3_ + *k*_−3_), which will be addressed under “Discussion.”

##### ADP Affinity Is Very Tight for WT MyHC-emb and R672C

The ADP affinity of myosin-S1 can be measured by preincubating S1 with varying concentrations of ADP and rapidly mixing with excess ATP to displace the ADP. A two-phase reaction is expected, consisting of a fast and a slow element representing the ATP binding to free S1 and ADP being displaced, respectively (Fig. 6*C*). Increasing the concentration of ADP does not alter the *k*_obs_ for the fast or slow phase but does affect the amplitudes (Fig. 6*D*) because more of the S1 is occupied by ADP that has to be displaced (slow phase) before ATP can bind. The measurement was repeated for each of the FSS mutations. However, the low fluorescence amplitudes observed on binding ATP made these measurements very difficult. The assay was only successful for R672C, and even then it was only possible to observe the loss of the fast phase. Using Equation 5, the ADP affinity (*K*_5_) could be determined; however, it was not significantly different from that of the WT MyHC-emb (Table 1). ADP displacement could not be measured for R672H and T178I due to a low signal upon nucleotide binding. Attempts to measure ADP binding using fluorescent nucleotides mant- and coumarin-modified ATP or ADP also result in no apparent change in fluorescence for R672H and T178I.

##### The V_max_ and K_m_ Values of the Three FSS Mutants Are Reduced

**FIGURE 7.**
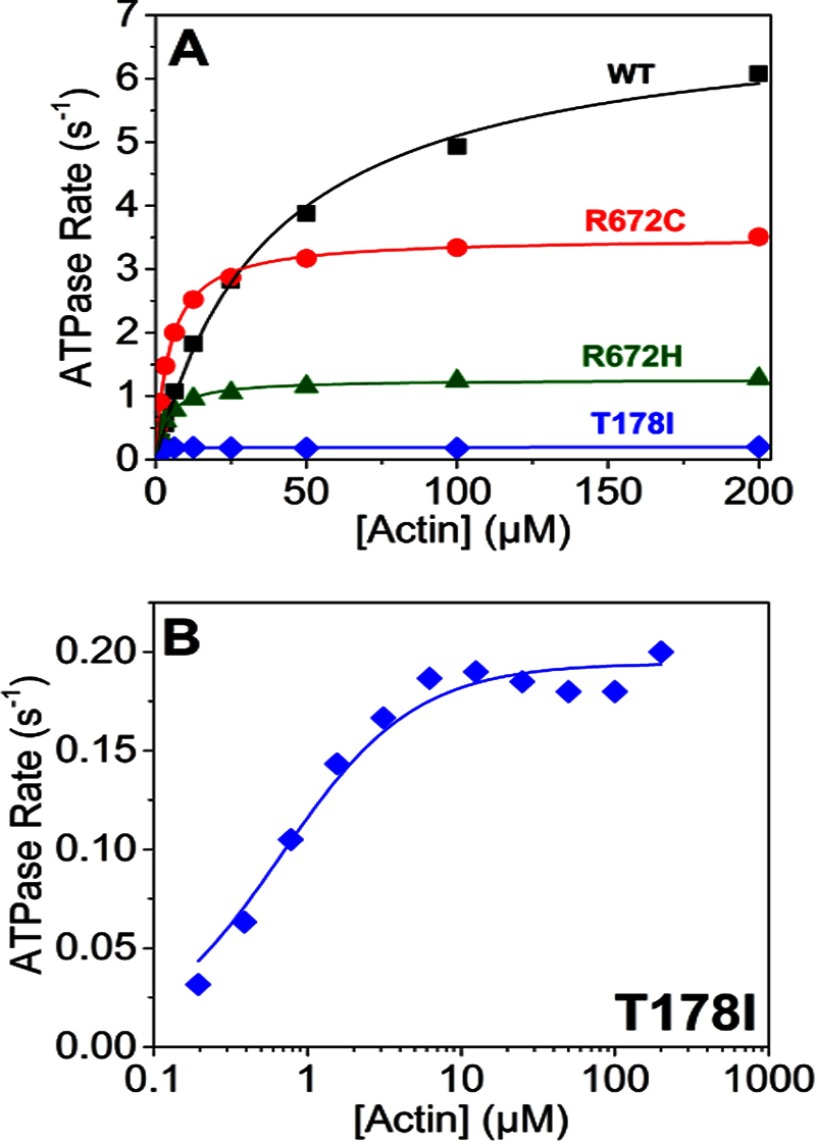
**ATPase assays of the WT MyHC-emb and the three FSS mutations.** *A*, actin activation of the S1 ATPases with best fit Michaelis-Menten curves superimposed on the data points. These fits gave a *V*_max_ = 7.0 s^−1^ for WT MyHC-emb and a *K_m_* = 38.5 μm (*filled squares*). The R672H (*filled circles*) had a *V*_max_ = 1.3 s^−1^ and a *K_m_* = 3.7 μm; R672C (*open squares*) had a *V*_max_ = 3.5 s^−1^ and a *K_m_* = 4.6 μm; and T178I (*open circles*) had a *V*_max_ = 0.2 s^−1^ and a *K_m_* = 0.7 μm. *B*, the ATPase assay of the T178I mutant on a log time scale to highlight the T178I fit to a Michaelis-Menten function despite a small activation by actin. Results plotted are from two protein preparations with 3–4 technical replicates each time.

The ATPase activity of the WT MyHC-emb and the three FSS mutants was measured as described under “Materials and Methods.” Both the values of *V*_max_ and *K_m_* are significantly greater for WT than those for the mutants (Fig. 7*A*). T178I data were plotted on a log [actin] scale (Fig. 7*B*) to highlight that there is activation along with the increase in actin concentration. However, the *K_m_* is so low and the *V*_max_ is so slow that this appears as a linear fit in Fig. 7*A*. The values of *V*_max_ and *K_m_* are summarized in Table 1 along with the *V*_max_/*K_m_*.

##### Homology Models of the WT MyHC-emb and Mutant Motor Domains Show Disrupted Interactions between the ATP Binding Pocket and Relay Helix in the Mutant Proteins

We examined the local structural interaction of the two mutated residues Arg^672^ and Thr^178^ using embryonic homology models based on both scallop myosin II crystal structures. These two residues are highly conserved throughout the myosin family and conserved in embryonic, cardiac, and scallop myosin.

The homology models of WT MyHC-emb show that residue Arg^672^ is buried in the core of the myosin molecule, located on the β3 strand of the central sheet. Arg^672^ interacts with residues on the two β-strands on either side (β2 and β4) and also with a residue located on the relay helix (Phe^490^). We will focus on two key interactions here, whereas a more detailed summary of interactions can be found in Tables 2 and 3.

**TABLE 2:**
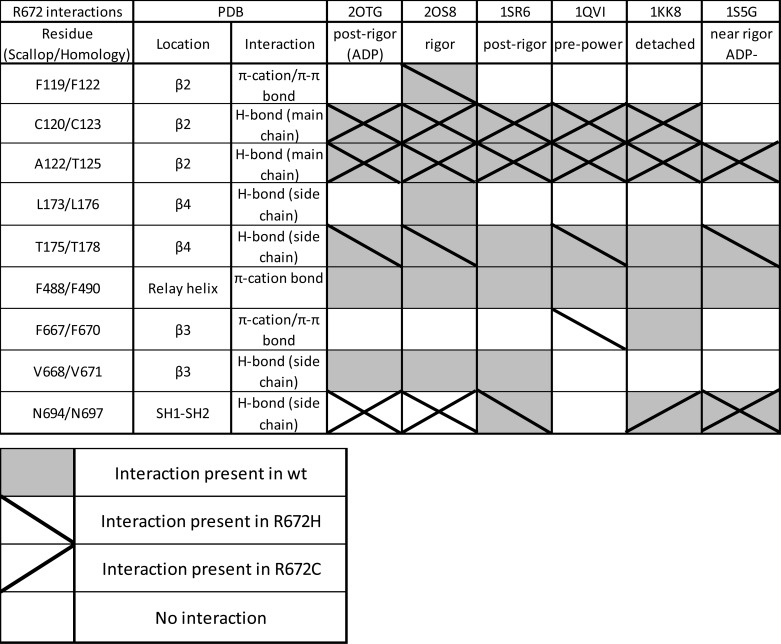
**Full list of interactions between Arg^672^ in WT MyHC-emb and the two Arg^672^ mutants** There are interactions between Arg^672^, on β3, and Cys^123^ and Thr^125^, on β2, of the central β-sheet in most conformations, the exception being the contact to Cys^123^ in the near rigor, ADP-bound state (PDB 1sS5gG). These interactions are predicted to be conserved in the R672C and R672H mutations. A π-cation bond that exists between Arg^672^ and Phe^122^ (β2) in the rigor state (2osOS8) is lost in both mutations, but a novel π-π bond is formed between R672H and Phe^122^ in the rigor state (20sOS8). The hydrogen bond between Arg^672^ and Leu^176^ (β1) is also lost in both mutations in the rigor state. Thr^178^ (on β4) hydrogen-bonds to Arg^672^ in each structure; however, this interaction is lost in the post-rigor (1srSR6) and the detached (1kkKK8) states for R672H and is lost completely for R672C. A second significant loss of interaction is the π-cation bond between Arg^672^ and Phe^490^ on the relay helix, which is non-existent in either mutation. A π-cation bond to Phe^670^ is lost for both mutations in the detached state; however, a π-π bond is formed in the pre-power state (1qviQVI). A hydrogen bond between Val^671^ is present in WT MyHC-emb in the rigor and post-rigor states (1osOS8, 2otgOTG, and 1srSR6) but lost in both mutations. The hydrogen bond between Asn^697^ on the SH1-SH2 domain is conserved in all three models in the near rigor ADP-bound state (1sS5Gg). However, this is lost in R672H in the detached state (1kkKK8) and in R672C in the post-rigor state (1srSR6). Interestingly, this interaction is formed in both mutants in the rigor (1kkKK8) and post-rigor (2OTGotg) states.

**TABLE 3:**
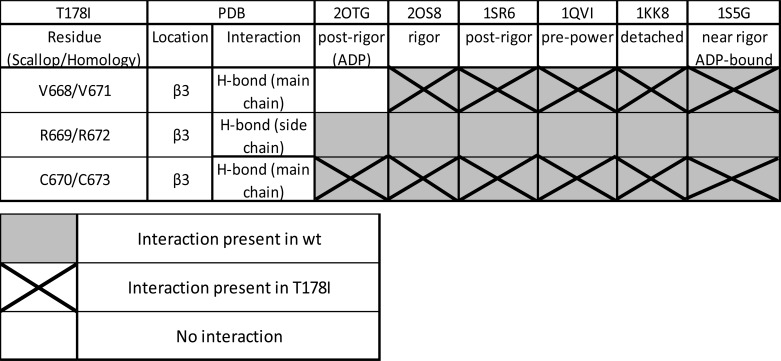
**Full list of interactions between Thr^178^ in WT MyHC-emb and T178I** The only change in the T178I mutation is the complete loss of the hydrogen bond between Thr^178^ and Arg^672^. The other two interactions between Val^671^ and Cys^673^ (both on β3) are both intact (except for Val^671^ in the post-rigor state, 2otg2OTG, which is not present in WT MyHC-emb or T178I).

One key interaction is the hydrogen bond between Arg^672^ and residue Thr^178^. Thr^178^ is located on β4 at the base of the P-loop, which is involved in ATP binding and hydrolysis. This Arg^672^-Thr^178^ interaction is found in all conformational states available for WT MyHC-emb, indicating that this hydrogen bond is preserved as the myosin molecule goes through the cross-bridge cycle. For both the T178I and R672C mutations, this hydrogen bond is lost in all myosin homology models, whereas for the R672H mutant, this hydrogen bond remains present in the majority of the structures.

Another key interaction is the π-cation bond between Arg^672^ and residue Phe^490^. Phe^490^ is located on the relay helix close to a bend in the helix that develops during the recovery stroke and recovers at some point during the power stroke. This interaction is present in all WT MyHC-emb homology models but is found to be consistently lost in both R672H and R672C mutations throughout the cycle. From our modeling, it is difficult to know if the loss of the hydrogen bond between the Thr^178^ and Arg^672^ in the T178I mutant results in disruption of the π-cation bond between Arg^672^ and Phe^490^.

Arg^672^, located on the central β-sheet, appears to facilitate interaction between the β4 strand and the relay helix. Note that Thr^178^ is at the end of the β4 strand and is immediately adjacent to the P-loop motif ^179^GESGAG^184^. This motif is one of three loops that bind to the γ-P_i_ of ATP. Loss of coupling of P-loop movements to the relay helix by mutation to either Arg^672^ or Thr^178^ is expected to cause major disruption of the cross-bridge cycle.

## Discussion

### 

#### 

##### WT MyHC-emb Has Properties of a Slow Type Myosin but Differs Significantly from the Classic Slow Type Myosin, MyHC-β

To our knowledge, this is the first complete kinetic analysis of the human embryonic myosin S1 isoform. Comparison of the kinetic properties of WT MyHC-emb with the slow WT MyHC-β (see Table 1 and Fig. 9) shows that there are significant differences between the two.

The high affinity of ADP for actin-myosin (*K*′_5_ = 14 μm) and the slow rate constant for ADP release from actin-myosin (*k*′_+5_ = 22 s^−1^) are indicators of a slow type myosin (40). The slow ADP release would result in a relatively long lived actin·myosin·ADP complex (lifetime 1/*k*′_+5_ = 45 ms *versus* 17 ms for MyHC-β under these conditions) that has high affinity for actin (*K*_DA_ = 0.53 μm) and therefore could hold tension against a load. The slow ADP release rate constant is likely to limit the maximum shortening velocity (*V_o_* = *d*/τ = working stroke/attached life time) to 5–10 nm/45 ms = 0.1–0.2 μm/s. At the same time, the ATPase *V*_max_ is similar for the two WT isoforms (6.0 and 7.0 s^−1^), resulting in a higher unloaded duty ratio for the embryonic myosin (duty ratio = *V*_max_/*k*′_+5_ = 0.33) compared with the β-myosin (0.10).

The second order rate constant for ATP binding to actin-S1 (*K*′_1_*k*′_+2_) is 2-fold faster for MyHC-emb than for MyHC-β. This is due to ATP affinity for actin-S1 (*K*′_1_) being almost 4-fold tighter (84 *versus* 327 μm) for emb-S1, whereas the maximum rate of actin dissociation (*k*′_+2_, 780 *versus* 1500 s^−1^) is half that of MyHC- β. This means that the detachment of actin is slower at saturating ATP but much less sensitive to the concentration of ATP available than the MyHC-β. This could be important because the MyHC-emb could maintain its activity in fluctuating ATP concentrations.

The fluorescence change associated with ATP binding to MyHC-emb S1 in the absence of actin is similar to that of β-cardiac S1 (39). The maximum *k*_obs_ for this process at saturating ATP concentrations is also 30% faster and close to that expected for a fast muscle myosin. This fluorescence change has normally been assigned to the steps controlling ATP hydrolysis and the recovery stroke (*k*_+3_ + *k*_−3_). ADP binding gave less than half of the fluorescence change for ATP binding to MyHC-emb, suggesting that the signal does primarily result from the change in the local environment of the conserved tryptophan at the end of the relay helix that is known to report the recovery stroke and hydrolysis step (41, 42). ADP affinity in the absence of actin is almost 4 times tighter for MyHC-emb than MyHC-β. However, in the presence of actin, the reverse is true. ADP affinity is almost 2-fold weaker for MyHC-emb. This results in a large increase in the thermodynamic coupling constant for ADP (*K*′_5_/*K*_5_), which increases almost 10-fold from 11.5 for MyHC-β to 95 for MyHC-emb. Thus, actin is much more effective at displacing ADP for MyHC-emb than for MyHC-β.

In summary, our data are consistent with MyHC-emb myosin being a slow type motor with a high nucleotide affinity, as recently described by Racca *et al.* (11). Our data also show that ATP binding (*K*′_1_*k*′_+2_) is 2-fold faster than that of MyHC-β, which is suggestive of a fast type myosin (43). However, the ADP release rate (*k*′_+5_) is almost 3 times slower than MyHC-β, which is indicative of a slow type myosin. Therefore, MyHC-emb properties sit between the fast and slow type myosins.

##### The Three FSS Mutations Significantly Slow the Cross-bridge Cycle Speed and Alter the Balance of the Cycle at Various Steps

The *V*_max_ for the ATPase cycle is reduced substantially (2–30-fold) for all three mutations with at least 10-fold increases in the apparent affinity of actin (*K_m_*). The rate constant for ADP release (limiting cross-bridge detachment) is little changed for T178I and R672C but reduced to one-third for the R672H mutant. This results in estimates of the duty ratio (equal to *k*′_+5_/*V*_max_) being reduced in each case from 0.3 for WT MyHC-emb to ∼0.18 for the two Arg^672^ mutations and very low for T178I (0.008). The two Arg^672^ mutation myosins would be able to sustain a lower force than the WT MyHC-emb, and T178I would be very ineffective as a force holding myosin, consistent with this mutation having the most severe phenotype (11).

The major differences between the WT MyHC-emb and the three mutations in the cross-bridge cycle are ATP-induced dissociation of actin from actin-S1 (*K*′_1_*k*′_+2_), ATP binding to S1 (*K*_1_*k*_+2_), and ATP hydrolysis (*k*_+2_ or *k*_+3_ + *k*_−3_) (see Table 1 and Fig. 9). The results suggest that ATP binding is slower than for WT MyHC-emb for all three mutations, by a factor of 2–5 for actin-S1 (*K*′_1_*k*′_+2_) and by 5–30-fold for S1 alone (*K*_1_*k*_+2_). The slowed rate constant for ATP binding is unlikely to have any physiological affect because the ATP in the cell is maintained at >1 mm, and each construct would be expected to bind ATP within 1 ms. The maximum rate constant of actin dissociation is, however, reduced 2–3-fold for the two Arg^672^ mutations, and this would result in a longer lived actin·myosin complex (2–3 ms).

One of the most striking changes is the reduction of *k*_+3_ + *k*_−3_ by 5–10-fold. If this represents the ATP hydrolysis step, then this would be a significant slowing of the ATPase cycle, leading to the myosin spending more time detached from actin in each ATPase cycle. This is highlighted in comparing the rate constant of the hydrolysis step (*k*_+3_ + *k*_−3_) with the *V*_max_ of the ATPase. For WT MyHC-emb, the hydrolysis rate constant is more than 20 times the value of *V*_max_, whereas it is reduced to 10- and 7-fold for the two Arg^672^ mutations.

A second point of interest is actin affinity with and without ADP present. In the absence of ADP, the actin affinities (*K*_A_) of the three mutants are significantly reduced compared with the WT MyHC-emb. However, because the experiments were conducted with 30 nm pyrene-labeled actin and the *K*_A_ values are <10 nm (except for R672H), we can only conclude that the affinities are very tight and may even be the same. We can, however, say that the affinity of R672H for actin is significantly reduced, almost 20-fold weaker. The affinity in the presence of ADP (*K*_DA_), on the other hand, is 1.5–4-fold tighter for all three mutants compared with the WT MyHC-emb. The only significant difference between the three mutants for ADP affinity in the presence of actin was the R672H, where *K*_5_ was 3-fold tighter.

From these data (Table 1), it is clear that all three of the mutations have significant kinetic differences compared with the WT MyHC-emb. The common difference between the three is the reduced apparent rate of ATP hydrolysis.

##### The Interaction between Arg^672^ and the Relay Helix Is Lost for the FSS Mutation

**FIGURE 8.**
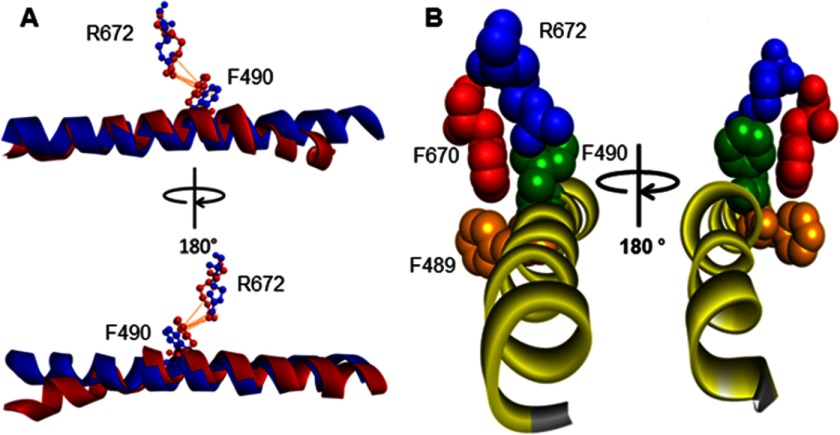
**Homology model of WT MyHC-emb showing the relay helix, Arg^672^, and Phe^122^, Phe^490^, and Phe^670^ in rigor and pre-power stroke.** *A*, structure based on scallop structure (PDB code 2OS8), where the motor domain is in the rigor conformation and the relay helix is straight (*blue*). There is a single π-cation bond between Arg^672^ and Phe^490^ shown in *orange*. A superimposed second relay helix (in *red*) is based on scallop structure in the pre-power stroke conformation (PDB code 1QVI). The relay helix is bent just after Phe^490^, and the π-cation interaction between Arg^672^ and Phe^490^ remains. *B*, Arg^672^ (*blue*), Phe^489^ (*orange*), Phe^490^ (*green*), and Phe^670^ (*red*) are shown with the relay helix to illustrate the phenylalanine residues acting as pivot point for the relay helix bend. The proximity of Arg^672^ to Phe^490^ and the interactions of Arg^672^ with Phe^490^ possibly anchor the helix, allowing it to bend.

This investigation was limited by the lack of tryptophan fluorescence change on ADP binding in the three mutants. Homology models for each mutation suggest a possible explanation for this loss of fluorescence. Arg^672^ and Phe^490^ (on the relay helix) form a π-cation bond, which is lost with the two Arg^672^ mutations. R672H has the potential to form π-π bonds (44), but none were observed in the homology models. These π-cation interactions form between positive ions or protein residues and the π face of a benzene ring or other aromatic structure (45). These interactions can be as strong as or even stronger than salt bridges, so a complete loss of such a contact could have a large effect on the protein. During the ATPase cycle, the C terminus of the relay helix bends away from the β-sheet, with the tryptophan immediately after the relay helix reporting a change in fluorescence as it moves. Homology models for the rigor-like conformation of myosin (PDB code 2OS8) and the pre-power stroke conformation (PDB code 1QVI) revealed that Phe^490^ is located just before the point in the relay helix where it bends as part of the power stroke/recovery stroke (Fig. 8*A*). Phe^490^ is one of three phenylalanines (Fig. 8*B*) that had previously been identified as part of a fulcrum on the relay helix in myosin S1 (46). This work also showed that Phe^489^ and Phe^490^ sandwich Phe^670^, forming the fulcrum that stays locked together through the whole transition. It is possible that the π-cation bond between Arg^672^ and Phe^490^ plays a structural role in holding the fulcrum together and maintaining contact with the β-sheet in which Arg^672^ sits.

Complete loss of this interaction in both Arg^672^ mutations could disrupt the structure of the fulcrum, making it less stable. This translates into a loss of amplitude in the tryptophan fluorescence signal, which indicates that the relay helix is not moving properly in the recovery stroke, and a slowing of the hydrolysis step linked to the recovery stroke. This complements the findings that the specific force of the R672C was reduced (13) because the conformational changes in the ATP and actin binding domains are not efficiently relayed to the converter domain and neck region.

A second important interaction is the hydrogen bond between Arg^672^ and Thr^178^. In the two mutations, R672C and T178I, this hydrogen bond is completely lost, and it is partially lost in the R672H. Destabilization of this region is the probable cause for the reduction in the *k*_+2_ or *k*_+3_ + *k*_−3_ step seen for all three mutations. Therefore, this bond between Arg^672^ and Thr^178^ must have some structural importance, bringing the residues required for hydrolysis into proximity with the ATP. The loss of this bond could account for the reduction in the ATP hydrolysis rate. The reduction in tryptophan fluorescence in the T178I mutant also suggests that the bond between Arg^672^ and Thr^178^ is having an indirect effect on the π-cation bond from Arg^672^ to the relay helix.

##### The FSS Mutations Slow Down the Dissociation Rate and, Once Detached, Spend Longer Not Bound to Actin, Leading to a Longer ATPase Cycle Time

**FIGURE 9.**
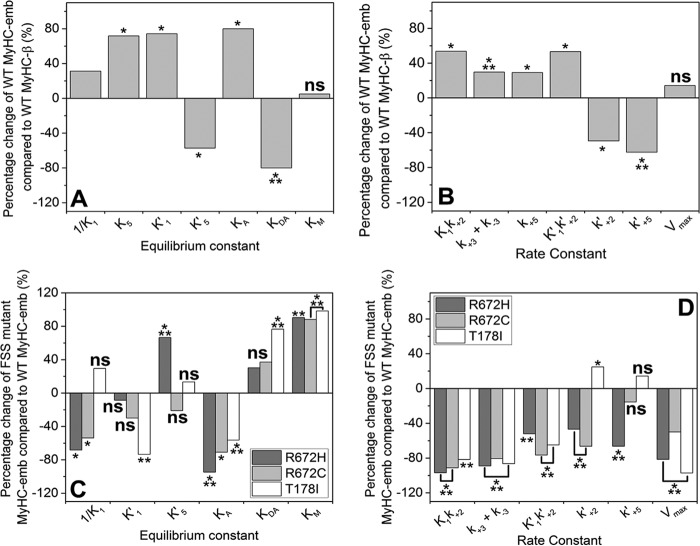
**Percentage changes in the rate and equilibrium constants of WT MyHC-emb compared with WT MyHC-β (*A* and *B*) and the three FSS mutations compared with WT MyHC-emb (*C* and *D*).** *A* and *C*, equilibrium constants are defined in M units; a positive percentage means an increase in affinity, whereas a negative value indicates a weakening of affinity. *B* and *D*, the rate constants are defined in units of s^−1^ or m^−1^ s^−1^, and a positive percentage indicates a faster rate constant, whereas a negative percentage indicates a slower rate constant. *ns*, not significant; *, *p* < 0.05; **, *p* < 0.01; ***, *p* < 0.001.

Our data support the finding that a longer, slower rate of relaxation as seen for FSS mutants could be explained by a reduced rate of detachment (13). There was a 2–5-fold reduction in the second order rate constant for detachment (*K*′_1_*k*′_+2_) for the three mutations (Fig. 9). However, the reduction in the ATP hydrolysis step that we have seen is a novel observation for the three FSS mutations. It is most likely that the destabilizing effects of the mutations on the P-loop are having a detrimental effect on ATP binding and ATP hydrolysis. Furthermore, the loss of interactions between the central β-sheet and the relay helix may hamper conformational changes within the molecule, leading to the actin dissociation step. Both would lead to a longer cycling time, where the myosin is bound to actin for longer or delayed in hydrolyzing ATP. This would leave the myosin mainly in the myosin·ATP or actin·myosin·ATP state, increasing the ATPase cycle time.

As the major myosin isoform expressed in embryonic development, the effects of the mutant alleles of embryonic myosin should manifest themselves during the formation of skeletal muscle and its earliest function. Just as with most myosin-based myopathies, the vast majority of reported cases of FSS are heterozygous, the prediction being that homozygous individuals would be inviable. It is noteworthy that FSS, a form of distal arthrogryposis, affects muscles in the lower legs and forearms, which appear to develop early relative to proximal skeletal muscle in fetal development (18). The timing and abundant expression of the mutant isoform during this stage could explain the symptoms seen post-birth, at a time when the expression of embryonic myosin has decreased significantly. Consistent with the joint contracture phenotype, these FSS mutations display severely reduced rate constants for cross-bridge detachment and slower cycling times. Understanding how the mutant myosin influences muscle contraction at different levels of expression along with the WT MyHC-emb myosin or an up-regulation of other myosins would be important to determine. We are currently developing models to allow prediction of the behavior of different myosin isoforms in a sarcomere (47, 48).

## Author Contributions

C. V. produced gene constructs, grew adenovirus, transfected cell lines to produce all cell pellets, and carried out steady state ATPase assays. J. W. purified proteins from cell pellets provided by C. V. and performed all transient kinetic assays except for the preliminary studies performed by M. J. B. L. L. and M. A. G. designed the study and supervised data collection. All authors contributed to the interpretation of data and writing and editing of the manuscript.

## Footnotes

4 The abbreviations used are:

MyHC: human sarcomeric myosin heavy chain
FSS: Freeman-Sheldon syndrome
PDB: Protein Data Bank.
